# Review of Cherenkov imaging technology advances in radiotherapy: single-photon-level imaging in high ambient light and radiation backgrounds

**DOI:** 10.1117/1.BIOS.1.2.020901

**Published:** 2024-07-18

**Authors:** Aubrey Parks, Jeremy Hallett, Alexander Niver, Rongxiao Zhang, Petr Bruza, Brian W. Pogue

**Affiliations:** a University of Wisconsin–Madison, Department of Medical Physics, Madison, Wisconsin, United States; b New York Medical College, Westchester, New York, United States; c Thayer School of Engineering at Dartmouth, Hanover, New Hampshire, United States; d DoseOptics LLC, Lebanon, New Hampshire, United States

**Keywords:** Cerenkov, radiation therapy, complementary metal-oxide-semiconductor, x-ray, filtering

## Abstract

**Significance:**

Single-photon-level imaging has been utilized for decades in closed dark environments; however, the utility for macroscopic imaging is more limited because it involves time-gating, filtering, and processing to view signals of interest. In radiation therapy delivery, a low-level signal called Cherenkov emission occurs from patients’ bodies, which is imaged with single-photon level sensitivity, mapping radiation dose deposition in tissue. Several key technological advances have been leveraged to make this extremely low-light signal overcome high background and noise in clinical settings.

**Aim:**

Our review summarizes specific technological advances that have led to a single-photon imaging in high radiation noise and high optical background environments possible. Our work discusses applications and future opportunities.

**Approach:**

Physical fundamentals of Cherenkov light, ambient room light, optical filtering, time-gating, and image processing are reviewed with key technological camera choices. This is followed by discussion of image quality, noise, and postprocessing, with current and future applications.

**Results:**

Invention and optimization of time-gating techniques and cameras with a single-photon capability were required to achieve real-time Cherenkov imaging. Requirements of video frame rate (≈10 to 30 fps), fast triggering (≈μs), clinically relevant spatial resolution (≈mm), single-photon/pixel sensitivity, and large field of view all led to intensified complementary metal-oxide-semiconductor cameras. Additional innovations in wavelength filtering, lens choices, and spatial and temporal postprocessing have allowed imaging that is not overwhelmed by ambient radiation noise or room lights. The current use provides real-time visualization of external beam radiotherapy on patient’s skin. Several emerging research areas may improve image quality and provide additional capabilities in biochemical sensing and quantification of delivery.

**Conclusion:**

The technical inventions and discoveries on how this light signal is sampled have led to real-time beam observation for dose delivery verification in settings where single-photon sensitive imaging is seemingly implausible while also opening the door to additional research applications.

Statement of Discovery Major technical advances in how to achieve single photon level imaging of Cherenkov emission have been outlined, including fast time-gating, online median filtering and frame averaging with background subtraction, and wavelength optimization. This optical time-gated imaging is the only methodology that exists today to image radiation dose delivery to human tissue in real time.

## 1 Introduction

Radiation has been used in medicine for over 100 years, and despite its current widespread use, a fundamental limitation in therapeutic delivery has always been the fact that x-rays are invisible to human vision. This limitation provides some uncertainty about the amount of damage delivered from radiation dose and complexity in the methods that are used to ensure its daily use and control. To mitigate this, the process of radiation dosimetry has developed into a mature science, but still the tools to quantify dose are limited to surrogates of *in vivo* dose. Most doses are estimated from measures of air exposure and often calibrated by measures in water or tissue-simulating phantoms, and there is essentially no direct or noninvasive way to directly quantify dose inside tissue. The discovery of Cherenkov light emission has been one of the very few emission signals from matter that shows the process of radiation dose delivery *in situ*. This Cherenkov light signal comes from the dose deposition and so is a direct linear correlate to the total dose delivered inside tissue,^1^ although the process of light exiting tissue is known to alter this. The technology to detect the Cherenkov light signals and the understanding of this light–tissue interaction is reviewed here.

### 1.1 Value of Cherenkov

Delivery of radiation from a linear accelerator as electron or x-ray beams is now widely adopted throughout cancer medicine with extensive control systems and a range of technologically advanced delivery and measurement systems. There are ∼4000 clinical linacs in the USA used for cancer treatment and ∼11,000 throughout the world.^2^ These devices are used to perform the majority of all radiotherapy, and >50% of all cancer patients receive it as part of their daily fractionated treatment course.^3^ The ability to deliver precision doses is thought to be exceptionally high, yet the reality is that delivery is not monitored at the patient. Direct validation of treatment doses is replaced by calculated estimates based upon calibration of the machine and computational treatment planning with verified patient setup. The value of Cherenkov imaging is that it shows a visualization of the dose on the patient’s skin,^4^^–^^6^ providing a real-time intuitive display of these dynamic treatments.^7^^,^^8^ The technology advances to capture this, and the implementation and interpretation of how these images are used is discussed here.

**Fig. 1:**
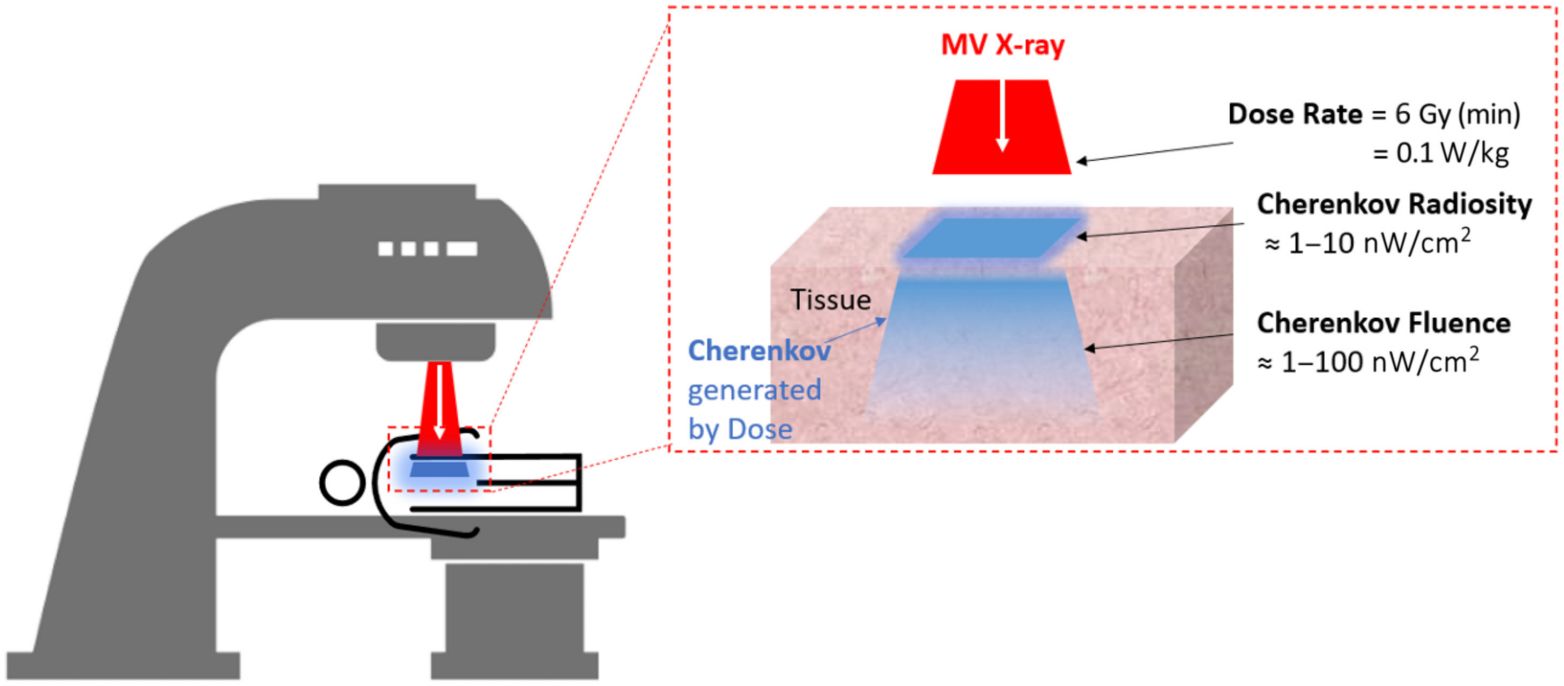
Image of x-rays entering tissue and the Cherenkov being emitted. Typical dose rate has Cherenkov radiosity of ≈1 to $10\;\mathrm{nW}/{\mathrm{cm}}^{2}$ and an internal Cherenkov fluence within tissue of ≈1 to $100\;\mathrm{nW}/{\mathrm{cm}}^{2}$.

The observed value of Cherenkov has been in seeing delivery incidents that should be fixed or small errors that need to be compensated for.^7^^,^^8^ An early prospective study showed 10% incident rate where Cherenkov could be utilized,^7^ although likely more realistic estimates throughout radiotherapy practice may be lower; however, because reliable tools to capture dose delivery do not currently exist, the true rate of errors is simply not well known. Most incidents are discovered through indirect means, and the investigation of them is time-consuming and expensive. Thus there is potential value in Cherenkov imaging for the fast, intuitive capture of all delivery to the patient. As the x-rays enter the tissue and induce soft electron collisions, the Cherenkov light is emitted and scattered within the tissue, and ultimately some fraction of it escapes from the tissue to be detected by the camera.^9^ The fact that tissue is translucent and emissive in the red to near-infrared wavelengths helps this process happen,^10^ as illustrated in Fig. 1.

### 1.2 Engineering Ultra-Low-Level Optical Signal Detection

Cherenkov is an extremely low-light signal emitted during soft electron collisions in the dose deposition process. The signal is perhaps 1% of the total dose delivered, and in radiotherapy the typical dose delivery rate is 6  Gy/min or 0.1  W/kg. A rough estimate of the Cherenkov yield might be 0.3  mW/kg of tissue. Converting this to water and assuming the signal comes from about 1 cm of tissue, this yields a rough unattenuated emission estimate of $0.3\;\mu W/{\mathrm{cm}}^{2}$.^11^ Thus the signal is low, and capture of this signal from several meters away is reduced significantly by the near isotropic emission. Imaging this level of light signal in a room with lighting on is exceptionally challenging and requires care in optical filtering,^12^ time-gating,^13^ and postprocessing,^14^ each of which are described below. The light signal is only able to be detected because the instantaneous Cherenkov light signal is above the room light’s instantaneous light signal, whereas the linear accelerator’s pulsing is off. The achievements developed for video-rate Cherenkov imaging are described here in terms of fast time-gated camera engineering, image processing, and associated optical and signal processing developments. Advances in optical engineering have been a critically important part of achieving Cherenkov imaging in practical radiotherapy use.

## 2 Optical Cherenkov Signals

### 2.1 Radiation Dose and Optical Cherenkov Emission

The local intensity of Cherenkov radiation can be estimated through the calculation:

$$I=\int_{E_{c}}^{E_{\max}}C(E)P(E)dE,$$ (1)

where $E_{c}$ is the threshold energy for Cherenkov radiation in a medium, which is 220 keV for biological tissue, C(E) is the number of Cherenkov photons emitted from a charged particle with kinetic energy of E per unit path length, and P(E) is the spectrum of the charged particles.^15^ Additionally, radiation dose is calculated through

$$D=\int_{0}^{E_{\max}}S(E)P(E)dE,$$ (2)

where S(E) is the mass stopping power of the medium at each energy. While the profiles of S(E) and C(E) are not equal, if P(E) is spatially independent, then the local intensity of Cherenkov emitted in tissue is proportional to the radiation dose.^15^ Below the threshold energy for Cherenkov emission, Cherenkov radiation’s absolute distance of travel is shorter than the continuous slowing down approximation range of electrons, meaning Cherenkov radiation will move less distance in tissue than electrons. Thus there will be a constant offset of the dose contributed by charged particles above the threshold energy to those below the threshold energy. The constant offset, along with P(E)’s spatial invariance, justifies the proportionality of local Cherenkov radiation to dose. Additionally, escaping Cherenkov photons experience a nearly Lambertian angular distribution, minimizing emission angle dependence for curved surfaces. Zhang et al. demonstrated the theoretical linear relationship of dose to Cherenkov radiation using both a Monte Carlo simulation of P(E) at various regions to validate its spatial homogeneity and experimentally imaging Cherenkov radiation emitted from megavoltage x-ray beams.

However, the linearity between absorbed dose and detected Cherenkov emission is distorted in patient imaging due to various tissue optical factors.^14^ Using Monte Carlo simulations on flat and cylindrical skin-like phantoms, Zhang et al. determined difference ranges between Cherenkov emission and radiation dose. Entrance/exit geometry and tissue optical properties contributed the largest variations of 50% and 20% variance, respectively, with beam energy, tissue curvature, and field size also contributing uncertainty. Thus, quantitative spatial frequency domain imaging was used to gather accurate mapping of tissue optical interaction coefficients to correct subcutaneous vasculature, interstitial blood, and pigment. In clinical trials, calibrated corrections were able to reduce vasculature effects slightly from 22% to 6% in one region and from 14% to 4% in another.^16^

Another method of correlating observed Cherenkov to absorbed surface dose uses x-ray radiodensity for macroscopic tissue types.^17^ Using a linear correlation between dose-normalized Cherenkov intensities and average CT number (HU) per patient, the $R^{2}$ linear regression coefficient between Cherenkov intensity and absorbed surface dose increased from 0.67 to 0.85 for a 6 MV beam and from 0.91 to 0.95 for a 10 MV beam. Thus it may be possible to calibrate Cherenkov intensity to dose, although more extensive validation is yet to be completed.

### 2.2 Intensity of Emission per X-Ray and Distance

**Fig. 2:**
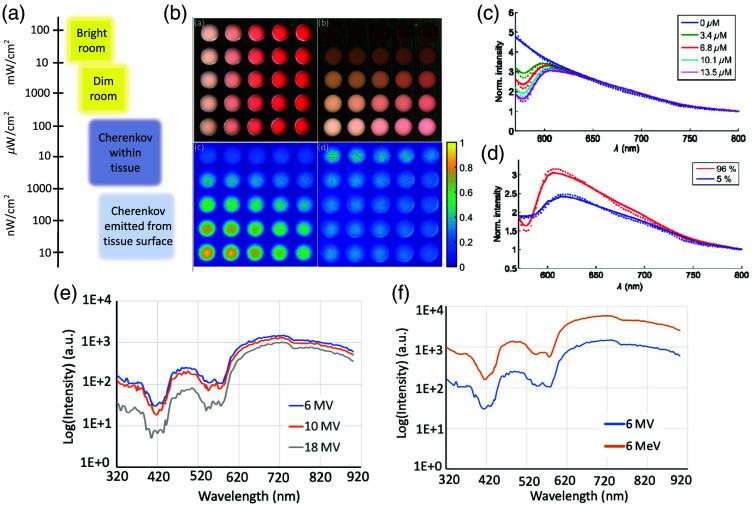
(a) Schematic of relative intensity of Cherenkov light, ranging with bright room light having the highest intensity of light, with Cherenkov emitted at tissue dimmer. (b) 1% intralipid and gelatin phantom with varying % blood concentrations in each column and 200  μm layers of varying melanin concentrations in each row. Detected Cherenkov due to irradiation by 6MV x-ray beam was imaged, and then normalized by the reflectance image in the gray channel. Displays altering intensity of Cherenkov detection due to blood and melanin concentration. Reprinted with permission from Ref. 14. (c) Increased blood concentration alters the pure $1/\mu^{2}$ Cherenkov spectrum by increasing absorption in the blue region while leaving the red region relatively stable. Reprinted with permission from Ref. 10 © Optica. (d) Increased blood oxygenation (${\mathrm{SO}}_{2}$) level (96%) increases the emitted spectrum in the red wavelength region Reprinted with permission from Ref. 10 © Optica. (e) Cherenkov emission spectrum shifts to be more blue with lower x-ray beam energy, and the overall signal increases due to deeper build-up region.^19^ (f) The spectrum is not significantly affected by beam type (electrons versus photons), but the overall yield is higher for electron beams.^19^

The emission of Cherenkov light from both megavoltage beams and radioactive decay is known to be relatively dim.^11^^,^^18^ During F18 decay, <0.006% of total energy released is Cherenkov emission, releasing ∼3  photons per decay over the 250 to 800 nm range.^18^ Glaser et al. determined the Cherenkov radiation rate in biological tissues to be on the order of 0.01 to $1\;\mathrm{nW}\;{\mathrm{cm}}^{-2}\;\mathrm{per}\;\mathrm{MBq}\;g^{-1}$. Additionally, the light fluence rate of Cherenkov radiation for radiotherapy beams in tissue was found to be 1 to $100\;\mu W\;{\mathrm{cm}}^{-2}\;\mathrm{per}\;\mathrm{Gy}\;s^{-1}$. In phototherapy applications, the total light fluence was on the order of $1\;\mathrm{nJ}\;{\mathrm{cm}}^{-2}$ for radionuclides and $1\;\mathrm{mJ}\;{\mathrm{cm}}^{-2}$ for radiotherapy beams.^11^ Although phototherapy may not be possible due to low rates of Cherenkov light fluence, diagnostic applications are reasonable for Cherenkov excitation of molecular probes. Figure 2(a) displays typical detected Cherenkov fluence values compared to typical room lighting conditions.

Real-time Cherenkov imaging requires optimization of the detection limits. Photon production, propagation, and detection are the key parameters.^9^ LaRochelle et al. determined that the distance from the source to the camera was the primary factor decreasing detection intensity due to the inverse square intensity dependence. The inverse squared dependence in detection dictates that the camera should be placed such that it is as proximal to the target as possible in order to maximize detected counts.^9^ Small camera to source distance, low f-number lens, and high quantum efficiency are required for sufficient light capture.

### 2.3 Tissue Effects and Cherenkov Spectrum

Tissue light absorption strongly effects Cherenkov intensity.^11^ In the absence of absorption, the fluence of Cherenkov light due to radionuclides is on the order of 5 to $500\;\mathrm{fW}\;{\mathrm{cm}}^{-2}\;\mathrm{per}\;\mathrm{MBq}\;g^{-1}$ and 4000 to $8000\;\mathrm{nW}\;{\mathrm{cm}}^{-2}\;\mathrm{per}\;\mathrm{Gy}\;s^{-1}$ for external radiotherapy beams.^11^ Figure 2(b) visualizes the effect of tissue composition on detected Cherenkov intensity, where both hemoglobin and melanin were varied to simulate attenuation in human tissues.

In the absence of absorptive materials, optical Cherenkov radiation exhibits strong emission of blue wavelengths and the number of photons per wavelength is proportional to $∼1/\lambda^{2}$, as described by the Frank–Tamm formula.^9^^,^^10^ The inverse squared relationship between wavelength and intensity in pure water spans from 300 to 1500 nm.^16^

At the location of dose deposition, the inverse square relationship holds, but as the light moves through the tissue, absorption and scatter impart spectral changes, modulating the signal.^10^ Specifically, the modulation arises from the optical absorption of water, lipids, and hemoglobin in tissue.^10^^,^^11^ The exact shape of spectral distortion is dependent on hardware factors, such as beam profile and distance to the detecting fiber, as well as biological factors, such as hemoglobin concentration and oxygenation affecting specific wavelengths. Axelsson et al. found that the prominent change in the spectrum with varying hemoglobin concentrations occurs at wavelengths shorter than ∼630  nm, seen in Fig. 2(c). An increase of hemoglobin oxygen saturation alters the spectral intensity resulting in a shift to the red region of the spectrum, demonstrated in Fig. 2(d).^10^ Similarly, Glaser et al. found that the fluence rate of emitted Cherenkov light in the presence of water, lipids, and hemoglobin was the strongest beyond 600 nm.

Changes to the spectrum of Cherenkov light emitted from tissue have additionally been investigated under varying beam type, incidence, energy, and field size using Monte Carlo simulation.^19^ Simulated beams of 6, 10, and 18 MV, 6 MeV, entrance and exit orientations, and field sizes of $1\times 1\;{\mathrm{cm}}^{2}$ up to $40\times 40\;{\mathrm{cm}}^{2}$ all show, effectively, the same spectral shape. When coupled to the quantum efficiency of red-optimized and green-optimized photocathodes, it was determined that there is a large increase in the detected Cherenkov with increasing field sizes, a slight increase in absorption with higher energy beams, and increased with electron beams compared to photon beams of the same energy.^19^ Figure 2(e) displays altering spectra according to beam energy, and Fig. 2(f) displays the varying spectra upon altering beam type.

## 3 Ambient Light Suppression

When compared to the ambient room light in a treatment room, the Cherenkov light emitted from the patient’s surface during external beam radiation therapy treatment is very dim. Commercial incandescent lights typically produce a surface irradiance in a room between 100 and $1\;\mathrm{mW}/{\mathrm{cm}}^{2}$.^13^ This signal strength is significantly higher than the irradiance of Cherenkov emission, which lies in the range between $1\;\mu W/{\mathrm{cm}}^{2}$ and $1\;\mathrm{nW}/{\mathrm{cm}}^{2}$ when produced by a typical linac beam.^13^ Due to this discrepancy, continuous wave detection of Cherenkov emission is essentially impossible to image with the room lights on.

### 3.1 Time-Gating Approach

**Fig. 3:**
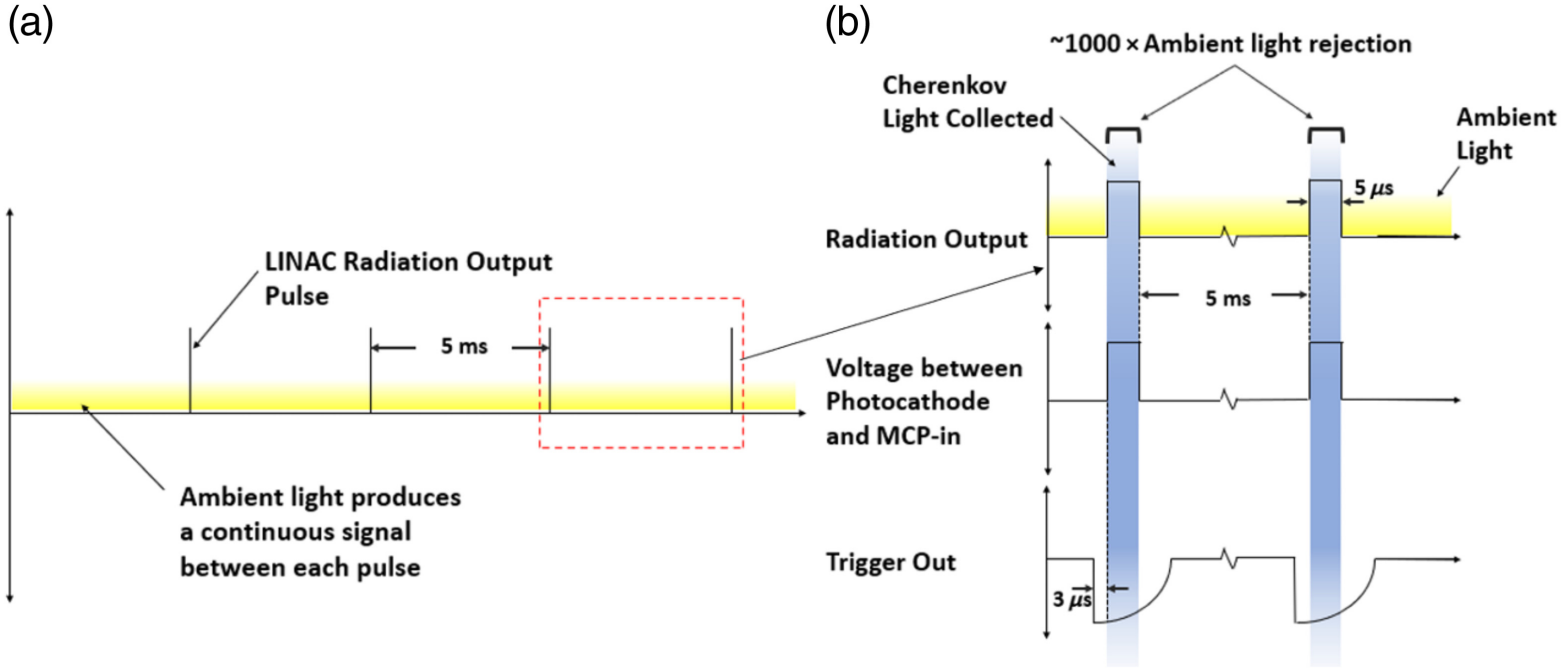
(a) The majority of the ambient light produced in the linac vault is continuous, and the duty cycle of the pulses is actually very low, around 0.1%, and so continuous imaging would largely just capture room light. (b) The timing scheme of a gated camera is shown, where a potential between the photocathode and the MCP input is applied during each trigger, leading to exposure only during the linac pulses.

By exploiting the pulsed nature of clinical linacs with time-gated cameras for Cherenkov detection, the ambient room light signal can be decreased by 1000×.^13^ Typical linac photon and electron beams are delivered in 3 to 6  μs bursts with a repetition frequency of 60 to 360 Hz,^20^ as illustrated in Fig. 3, correlating to a duty cycle of roughly 0.1%. By only exposing the Cherenkov camera sensor during the pulses, the ambient light signal is reduced to the same order of magnitude as the Cherenkov signal.^13^ Background images collected between pulses are normalized and subtracted from the pulse images to remove any remaining room light signal.^21^

In Ref. 13, the optical signal spectra from the Cherenkov emission was captured using an intensified charge-coupled device (CCD) camera (PI-MAX3 RB Gen II, Princeton Instruments, Acton) coupled to a 13 m optical fiber placed in the center of the linac beam. The PI-MAX camera’s acquisition was gated to the linac pulses through an analog trigger signal fed through a BNC cable from the linac. An ambient light spectrum was collected with identical acquisition timing without any linac pulses, which was then subtracted from the original beam gated spectra. Resulting data showed the spectrum of Cherenkov light, demonstrating the feasibility of imaging weak Cherenkov emission during radiation therapy while leaving the room lights on for patient comfort and safety.^13^

**Fig. 4:**
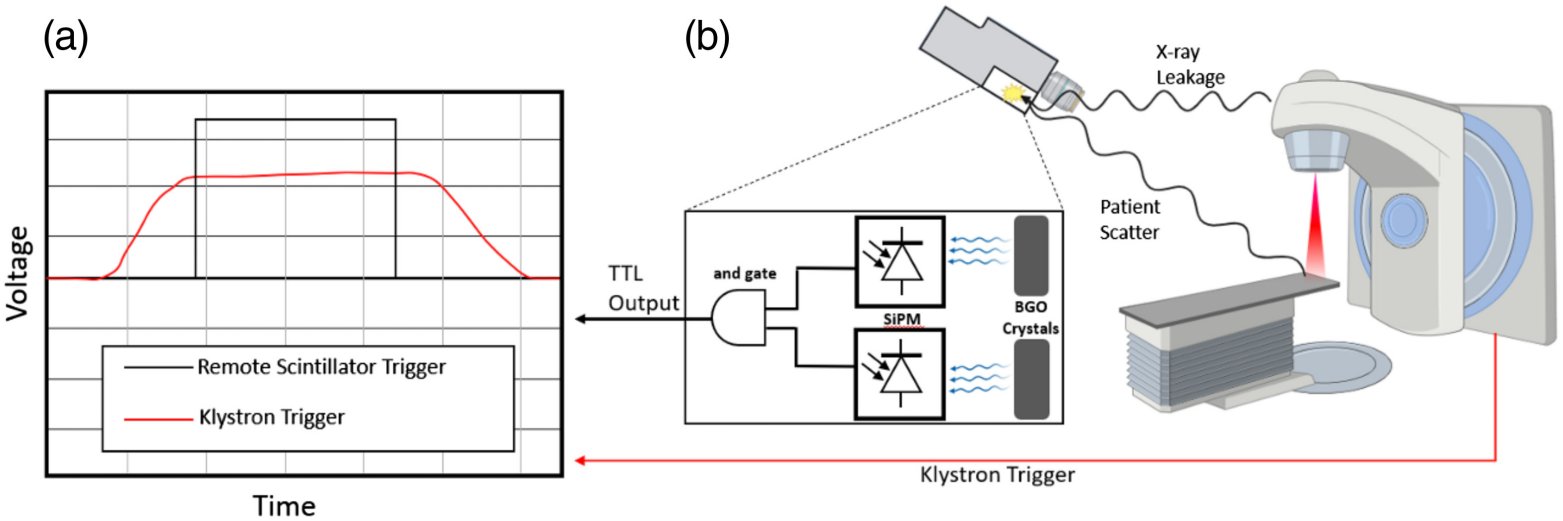
(a) The signal produced by the scintillator crystal coincidence system matches the time structure of the wired klystron signal coming from the linac. The success in linac pulse detection using the remote scintillator signal eliminates the need for a wired connection. (b) The scintillators detect leakage and scatter x-rays, emitting light detected by an SiPM.

Early measurements utilized the Klystron voltage (KlyV) or the target current signal port (TargI) as direct electrical line triggering from the linac control unit for camera intensifier gating.^20^ However, electronic feedback in the BNC cables along with manufacturer restrictions limit the practicality of wired triggering. Ashraf et al.^20^ developed remote trigger gating, which leverages the scatter and leakage photon signal detected by a silicon photomultiplier (SiPM) tube coupled with a scintillator detector to gate the image intensifier, as illustrated in Fig. 4. The SiPM’s current signal was converted with a current-to-voltage amplifier to act as a trigger and was saturated, regardless of how far from the linac the camera was placed in the room.^20^

The remote system resulted in a cumulative ROI intensity increase of roughly 1.5% over the cabled system and the average pixel intensity was found to be 0.9% greater on average for the remote triggering, increasing both the practicality of use in the clinical setting and Cherenkov signal.^20^ The study also addressed trigger activation from other radiation sources (such as cosmic rays, neutron activated products, and scintillator radioactivity) by connecting two detectors for coincidence gating with an AND gate, effectively suppressing false positive beam detection.^20^ Modern C-dose Cherenkov imaging cameras employ a similar remote triggering unit to distinguish the false scatter radiation events from the beam pulse.

### 3.2 Spectral Filtering

Cherenkov imaging can be used in high-dose rate Brachytherapy QA^22^ and cyclotron-based proton treatments.^23^ However, as these modalities have high repetition rates approximating continuous source treatments, it is not practical to gate the camera intensifier to the treatment duty cycle. Additionally, captured ambient room light remains a prevalent source of image quality degradation in EBRT. Thus spectral filtering light suppression might be necessary to improve Cherenkov imaging for continuous radiation treatments.

**Fig. 5:**
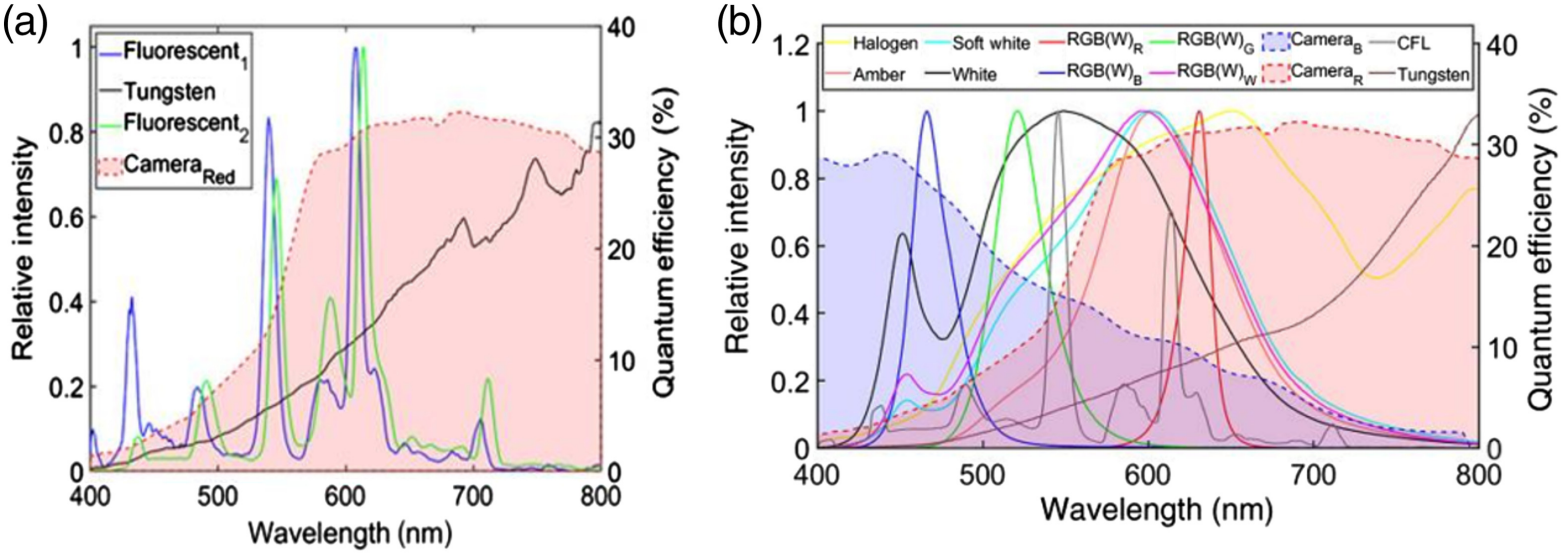
(a) The camera generally used for Cherenkov imaging has a quantum efficiency weighted in the red wavelength region. This allows for the efficient collection of much of the ambient room lights. (b) Some replacement room light choices fall outside the range of the red wavelength collected by the camera. Reprinted with permission from Ref. 12.

Rahman et al.^12^ investigated the spectral properties of Cherenkov emission with various fluorescent and LED lights. Lights already present in the linac vault in the Dartmouth-Hitchcock Medical Center Oncology Department, including two fluorescent lights and an emitting tungsten source, with spectra shown in Fig. 5(a), were compared to various possible replacement lights.^12^

Because the Cherenkov spectrum exiting patient tissue falls in the red wavelength range and the Cherenkov camera is optimized for this region,^10^ blue LED ambient room lights are optimal, as demonstrated in Fig. 5(b). However, this can be an uncomfortable lighting situation for patients. White light LED sources coupled with a 675 nm filter produces comparable results to a purely blue light with a color temperature of 6000 to 6500 K more closely matching the typical temperatures found in room lights (2500 to 6500 K).^12^ LED lights can produce a flicker in real-time Cherenkov imaging due to the repetition rate of the linac and LEDs, although constant-current LED drivers can eliminate this.^12^

## 4 Technology for Fast Image Capture

### 4.1 Time-Gated Intensified Cameras

*In vivo* surface beam monitoring with Cherenkov emission requires optimized camera hardware for qualitative and quantitative images.^24^ There are many options for cameras for low-light imaging, including sensors that are composed of (i) complementary metal-oxide-semiconductor (CMOS), (ii) CCD, and (iii) single-photon avalanche detector (SPAD) arrays. There are electron multiplying EM versions of the first two sensors, and both can be coupled with an image intensifier (II) at the front end. The choice of sensor and technique is driven by background light suppression and fast frame rate with the need for video rate acquisition. There is necessity to have amplification between the incoming light and the readout stage, accomplished using an image intensifier coupled CMOS or CCD camera.^24^

**Fig. 6:**
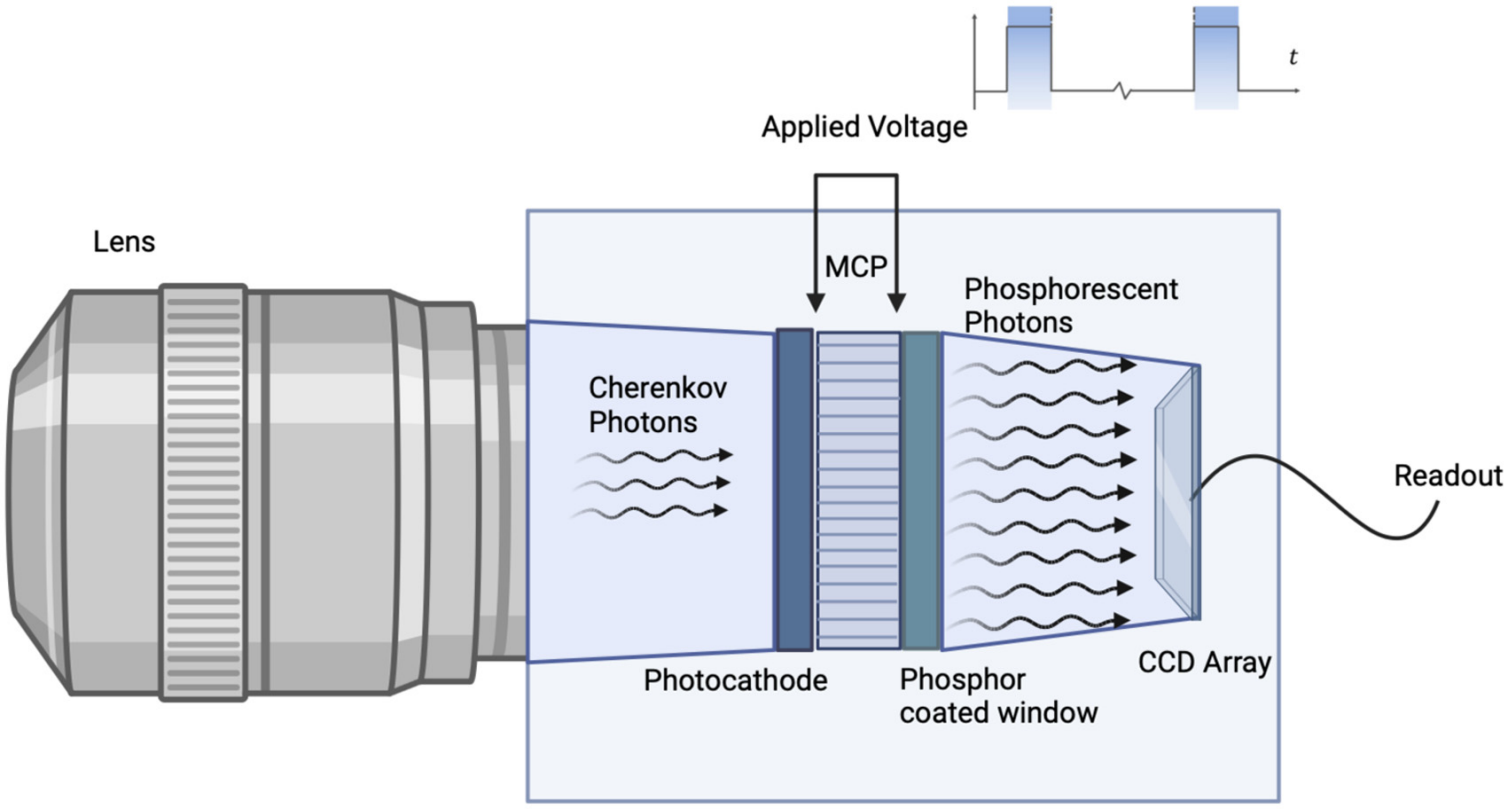
Typical intensified camera interior. Cherenkov light is captured by the lens and focused onto the photocathode, which emits an electron image into the MCP through a low applied voltage over the photocathode and phosphor coated window. These electrons are accelerated through the MCP by the high voltage, bouncing off the dynode walls, and multiplying the electron signal within each channel. This multiplied electron image from each channel ends at a phosphor coated exit window, which emits phosphorescent optical photons. These luminescent photons are focused onto a sensor array (either CCD or CMOS) through either an imaging fiber taper or lens coupling, turning the optical signal back into an electron image with amplification in each pixel. The applied high voltage on the MCP is synced with the time-gating of the signal expected, such that when the linac beam is on, the MCP high voltage is applied, and when the linac beam is off, the MCP applied voltage is off, suppressing image capture during linac off periods.

This solution produces a functional system with many advantages. However, intensifiers have limitations in terms of moderate dynamic range and high cost. Additionally, these intensifier microchannel plates (MCPs) are limited in the maximum spatial resolution by the number of lateral channels, which is usually much less than the number of sensor pixels.^25^ Thus, while intensified cameras are a valuable solution for low-intensity Cherenkov imaging, future innovations in optical sensors could lead to superior results or reduced cost (Fig. 6).

### 4.2 Single Photon Avalanche Diode and Quantum Sensors

A possible alternate single-photon-level sensor for this application is the SPAD, which have become increasingly popular for biophysics and biochemistry applications due to their single-photon sensitivity, picosecond response, and improved spatial resolution.^25^^,^^26^ Unlike intensified CMOS and CCD cameras, large CMOS SPAD arrays demonstrate high timing performance with no global count limitations creating potential for multichannel single-photon counting with parallel readout and fast data processing.^25^

**Fig. 7:**
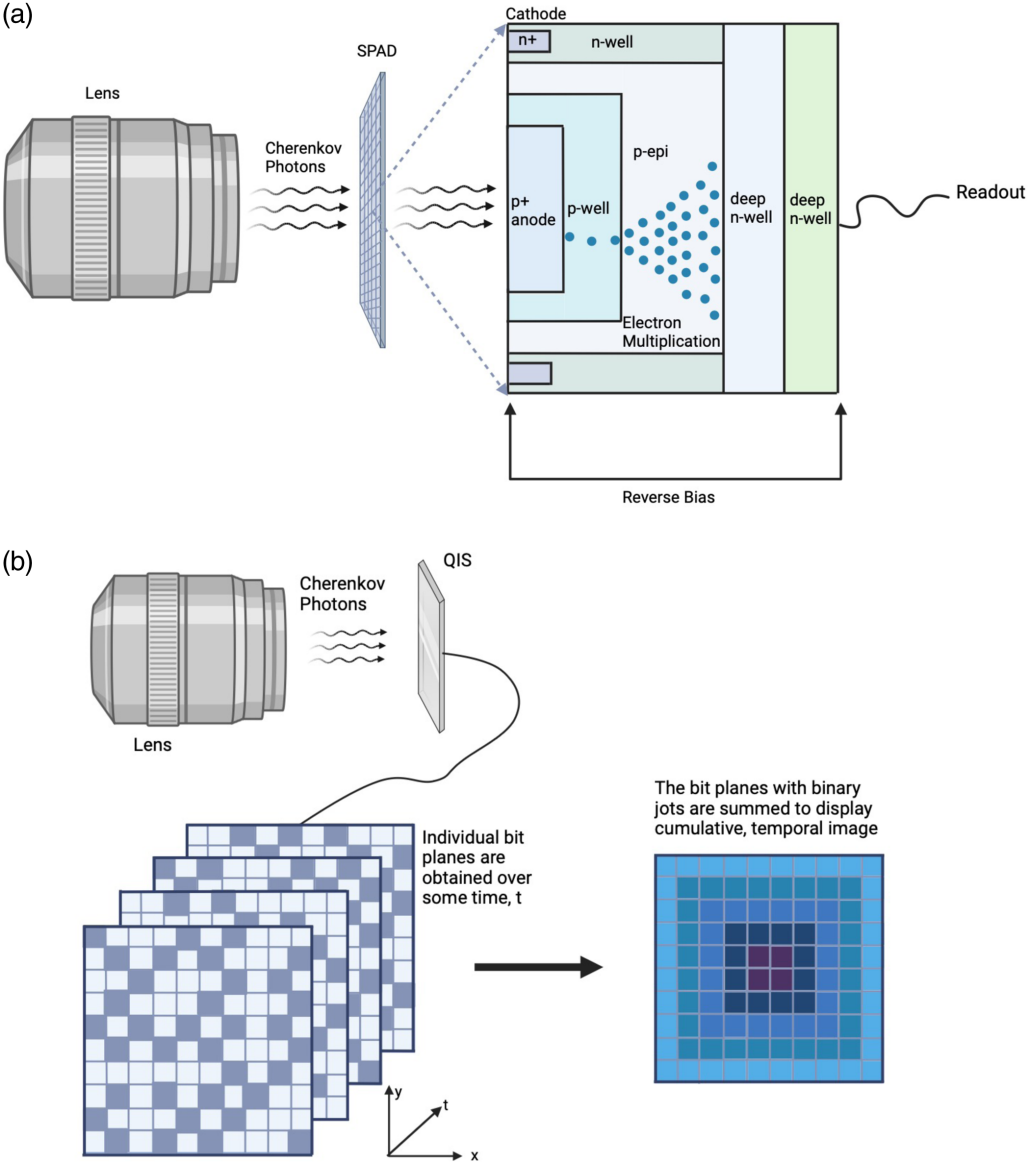
Alternate potential solutions for time-gated multipixel sensor Cherenkov imaging. (a) Typical setup for a SPAD imaging sensor. The Cherenkov light is focused by the lens onto the SPAD array. In each pixel of the array, there is a reverse biased p-n junction on a photodiode. Photons incident into the active area produce electron-pairs, triggering an avalanche effect in the multiplication region of each pixel. After readout, the avalanche is quenched by lowering the reverse bias voltage below the breakdown voltage. (b) Typical QIS acquisition setup. As Cherenkov light moves through the lens, it is incident on the QIS array, where individual binary bit planes are obtained over some time. The individual bit planes are pixels and an SPAD array and represent the binary image at one point in time. As the individual bit planes are summed, a cumulative image is displayed, representing the entire temporal image that was obtained.

Each SPAD pixel is a photodiode that is reverse biased above its breakdown voltage.^27^ When incident on the active device area, photons produce electron–hole pairs triggering an avalanche of secondary carriers, creating a self-sustaining amplification, which is then quenched by lowering the SPAD bias voltage below the breakdown voltage.^27^ Before the next photon can trigger an avalanche, the voltage is restored above the breakdown voltage.^27^ Figure 7(a) demonstrates the function of an SPAD array. After sufficient exposure, each row is sequentially read into a subarray, erasing the memory of the previous row.^25^ Thus, the internal sensor communicating the time between row readouts determines the overall readout speed.^25^ Just as with intensified detectors, digital SPAD imagers use a time-gating technique to maximize light detection. Photons will only trigger an avalanche if they arrive during the open gate on time.^25^

In order to improve image quality, specified by signal-to-noise ratio and dynamic range, there is a trade-off with the fill factor.^26^ Factors to take into account for these cameras are the pixel isolation, pitch, fill factor, photon detection probability, photon detection efficiency, dark count rate, afterpulsing, and timing jitter performance.^26^

More generally than the previous sensors, the concept of a quanta image sensor (QIS) was conceived in 2005 as the next major paradigm in solid-state sensors.^28^^,^^29^ Through extreme miniaturization of pixel sizes in CMOS sensors, increased storage capacity, and expanded digital processing, the goal of QIS is to optimize single-photon event capture in ultralarge pixel arrays and provide images through intensive postprocessing. QIS’s comprise an array of subdiffraction limit binary sensor pixels. Each of these binary pixel values is read as a logical output of 1 or 0 corresponding to the presence or absence of a photon strike at a location in the incident field with a goal of 1000 fields read per second. Figure 7(b) displays a typical setup for a QIS acquisition. By locally processing the pixel “jot” data, postcapture at arbitrary pixel sizes, time-sequencing or integrations are all possible.^29^ The small pixels provide increased resolution, extended low-light sensitivity, and enhanced photon-number-resolving capability over a standard CMOS camera.

**Fig. 8:**
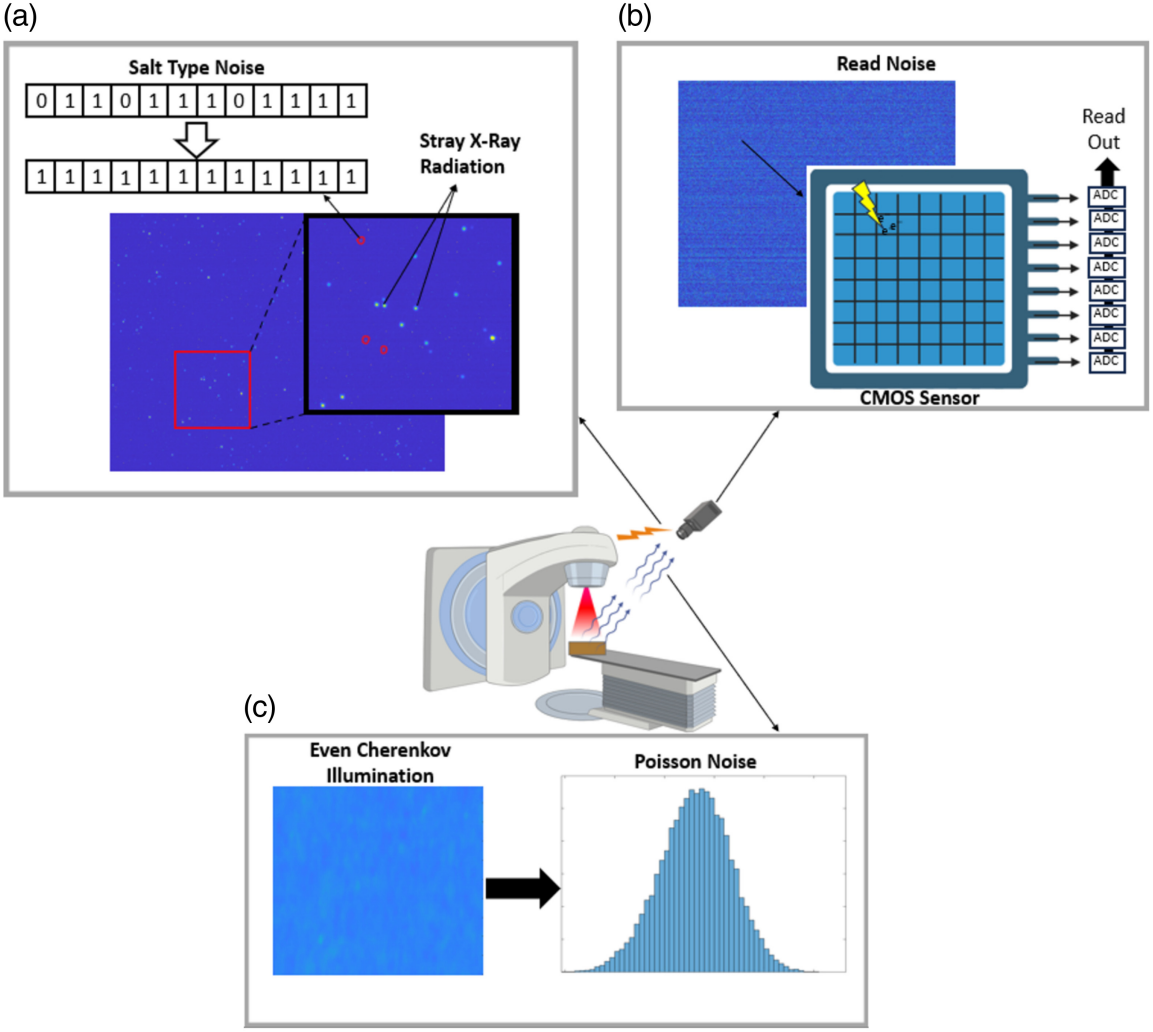
(a) Bright spots in Cherenkov images result from stray radiation coming off of the linac head, which can also lead to single-pixel readout errors manifesting as either random or permanent bright pixels salt noise. (b) Fixed pattern noise is a result of the detector architecture where all detector elements in a row share an ADC for readout. (c) Because of counting statistic properties, evenly irradiated regions of Cherenkov images produce a Gaussian spread of pixel intensities. Additionally, the read noise and dark current contribute to this Gaussian spread.

Cherenkov imaging was tested with the concept of QIS using a SPAD sensor^30^ where dose delivery was first imaged in water to validate the signal linearity. By gating the SPAD with the linac x-ray beam pulses, Cherenkov radiation was imaged from a patient during treatment, registering 1 to 2 photons per pixel above ∼1  photon per pixel background.^30^ Maximum Cherenkov radiance values were observed to be ≈3 to $4\times 10^{9}\;\mathrm{ph}\cdot {\mathrm{sr}}^{-1}\cdot {\mathrm{cm}}^{-2}\cdot s^{-1}$ for beams incident on a patient, corresponding to average radiant exitance of 2 to $3\;\mathrm{nW}/{\mathrm{cm}}^{2}$.^30^ In theory, the SPAD could outperform the best ICMOS by a factor of 50 when comparing the noise power density for a given geometry.^30^ However, while these SPADs can image Cherenkov with high sensitivity, the major limitation today is the low frame rate per linac pulse (4 to 5  μs each), which ultimately limits their use. This is because it means that relatively little of the available Cherenkov is captured in each linac pulse, ultimately lowering the detected signal because of camera deadtime. Thus the current time sequence of capture today would need to be improved for them to be viable options for Cherenkov imaging (Fig. 8).

### 4.3 Conclusion

The low-intensity Cherenkov emission from tissue poses a challenge for imaging techniques. ICCD cameras are beneficial to use due to their high resolution, good sensitivity, short acquisition times, and a large field of view.^31^ However, they have a low dynamic range, the MCPs are limited to a maximum achievable global count rate, and they are generally costly.^25^ SPAD cameras, conversely, have single-photon sensitivity, picosecond response, good spatial resolution, fast data processing, high timing performance, although are limited by frame rate mismatch with the linac pulses and a low fill factor in the camera.^25^ Ultimately some better sensor with SPAD or QIS capabilities for single-photon sensing with elimination of amplification-noise, efficient x-ray noise rejection, fast readout speeds, and decreased complexity would be ideal.^28^^–^^30^ For optimal Cherenkov imaging, the fast time-gating needs to match the pulse length and duty cycle of the linac and provide high signal-to-noise ratio with mm level spatial resolution. As of now, the ideal sensor for these characteristics remains the ICMOS option; however, it is quite possible that low noise sensors with fast electronic shutters may ultimately compete with this in the future.

## 5 Image Quality

### 5.1 Typical Cherenkov Noise and Clutter

The presentation of noise in the raw video frames of Cherenkov imaging is typical for CMOS camera systems, including noise associated with the camera system itself, the input signal, and stray radiation from the linac head. The stray and signal related noise are stochastic in nature and independent of the camera hardware, but the camera design heavily influences the camera related noise.^32^ These sources of noise are an ongoing challenge for Cherenkov imaging and development.

#### 5.1.1 Camera related noise

In a CMOS camera, when photons interact with each pixel, they produce electrons, and an amplifier and capacitor convert the charge produced into voltage. Each column of pixels has its own analog to digital converter (ADC), allowing for high-speed imaging. This fast readout architecture, however, causes read noise through random variation in the readout process.^32^ Although CCD cameras typically contain a single preamplifier, which converts the charge to a voltage for all pixels producing a Gaussian spatial noise distribution, CMOS sensor read noise spatially experiences a non-Gaussian, skewed distribution due to the individual readout structure for each pixel. This noise is often specified through the median and root mean square values of the distribution measured as the number of electrons.^32^^,^^33^ Additionally, read noise increases with faster readout speeds.^33^ Gating to the pulse of a linac for Cherenkov imaging requires fast readout and acquisition leading to higher noise levels. Read noise is also proportional to the input capacitance of each pixel, according to Eq. (3), where B and T are the circuit’s bandwidth and temperature, respectively. Therefore, lowering the input capacitance can have a large impact on the noise:^34^

$$\sigma \propto C\sqrt{B\times T}.$$ (3)

Additional read noise artifacts manifest as horizontal streaking across the image. The readout electronics in CMOS sensors are organized such that each column of the image shares a single ADC. Therefore, slight spatial variations in ADC performance result in these artifacts.^32^^,^^35^

CMOS sensors also experience noise produced from data transmission, memory errors, and errors in the digital to analog conversion.^36^ Salt and pepper noise is a pattern of noise where some pixel values become corrupted, getting set to either a maximum value of 255, for an eight-bit system, or 0,^37^ creating large contrast relative to the surrounding pixels.^38^ Salt and pepper noise can be mathematically represented in the following way:

$$n(i,j)=\{\begin{array}{ll} s_{\min} & P(n)=p\\ s_{\max} & P(n)=q\\ u(i,j) & P(n)=1-q-p \end{array},$$ (4)

where $s_{\min}$ and $s_{\max}$ are the minimum and maximum possible pixel values; p and q are probabilities that a pixel will be set to the minimum and maximum values, respectively; n(i,j) is the noisy image; and u(i,j) is the image free of salt and pepper noise.^36^^,^^39^ Several methods exist for removing salt and pepper noise from images, notably, median filtering.^40^^,^^41^ Typically, the CMOS sensors employed in Cherenkov imaging only experience salt type noise caused by stray radiation depositing extra charge into a pixel or, more commonly, permanently damaging the pixel such that it always reads out a specific value.

#### 5.1.2 Signal related noise

Photon shot noise, resulting from the statistical nature of the photon flux incident on the image sensor, is the dominant source of noise in high signal regions of an image.^32^^,^^42^ The inherent quantum uncertainty in the emission of photons and the statistical uncertainty in whether the photon will interact in the detector material causes a noise pattern following Poisson statistics.^32^ Thus the noise standard deviation is proportional to the square root of the mean of number of detected photons $p_{\text{in}}$ times the average gain factor of the intensifier (G). The proportionality constant is contingent on the inherent dynamic range of the intensifier (F):^32^^,^^43^^,^^44^

$$\sigma_{\text{shot}}=G\times F\sqrt{\left(p_{\text{in}}\right)}.$$ (5)

It is believed that this distribution for photon counting holds for Cherenkov signal.^45^ Because this source of noise is inherent to light detection, it cannot be removed through optimal camera design, however, larger pixel binning, sacrificing the spatial resolution of the image, can provide suppression.^43^

In addition to noise from counting statistics, individual photons striking the CMOS sensor create a footprint of data that spans a radius of pixels, which is essentially a Gaussian probability distribution of where the photon interacted on the intensifier.^46^ The spread of pixel counts from a single photon occurs due to the resolution loss at the phosphor and intensifier output window causing the intensified signal to spread, illuminating several pixels.^47^

#### 5.1.3 Stray radiation noise

Additional signals exist within the linac bunker, including stray radiation escaping from the linac head or scattering off the patient, which interact with the CMOS sensor and produce hot pixels, adding to inherent noise.^31^^,^^46^ This source of noise is effectively suppressed in Cherenkov imaging using a combination of temporal and spatial median filters.

Although stray radiation noise sources are well understood, their superposition and background subtraction in the single-frame image processing leads to a complicated pattern of image mottle. This pattern is difficult to characterize and therefore makes the reduction of statistical noise a challenging task.

#### 5.1.4 Image filters

As already mentioned, stray radiation as well as salt type noise can be mitigated with spatial and temporal median filters. Spatial medium filtering is defined by a moving window averaging out a spatial region of pixels. Likewise, temporal medium filtering compares sequential frames and averages their pixel values.^48^ This processing suppresses bright pixels that do not correlate with image structures that are more constant in time and space. This processing can greatly improve the overall image quality; however, it leaves behind a mottle pattern, which can be difficult to model. Further, noise reduction methods must be studied to continue improving Cherenkov image quality.

### 5.2 Detective Quantum Efficiency Assessment

Several methods can be investigated to remove noise and clutter from Cherenkov imaging, including postprocessing, new acquisition techniques, and improved hardware. However, to properly compare Cherenkov images, a quantitative metric is needed. Detective quantum efficiency (DQE) is fundamentally tied to image quality in the field of radiography, describing the ability of a detector to create an image out of incident x-ray photons.^49^ Alexader et al.^50^ influenced the development of the Cherenkov cameras by measuring the DQE of the Gen3 and Gen2+ systems.

**Fig. 9:**
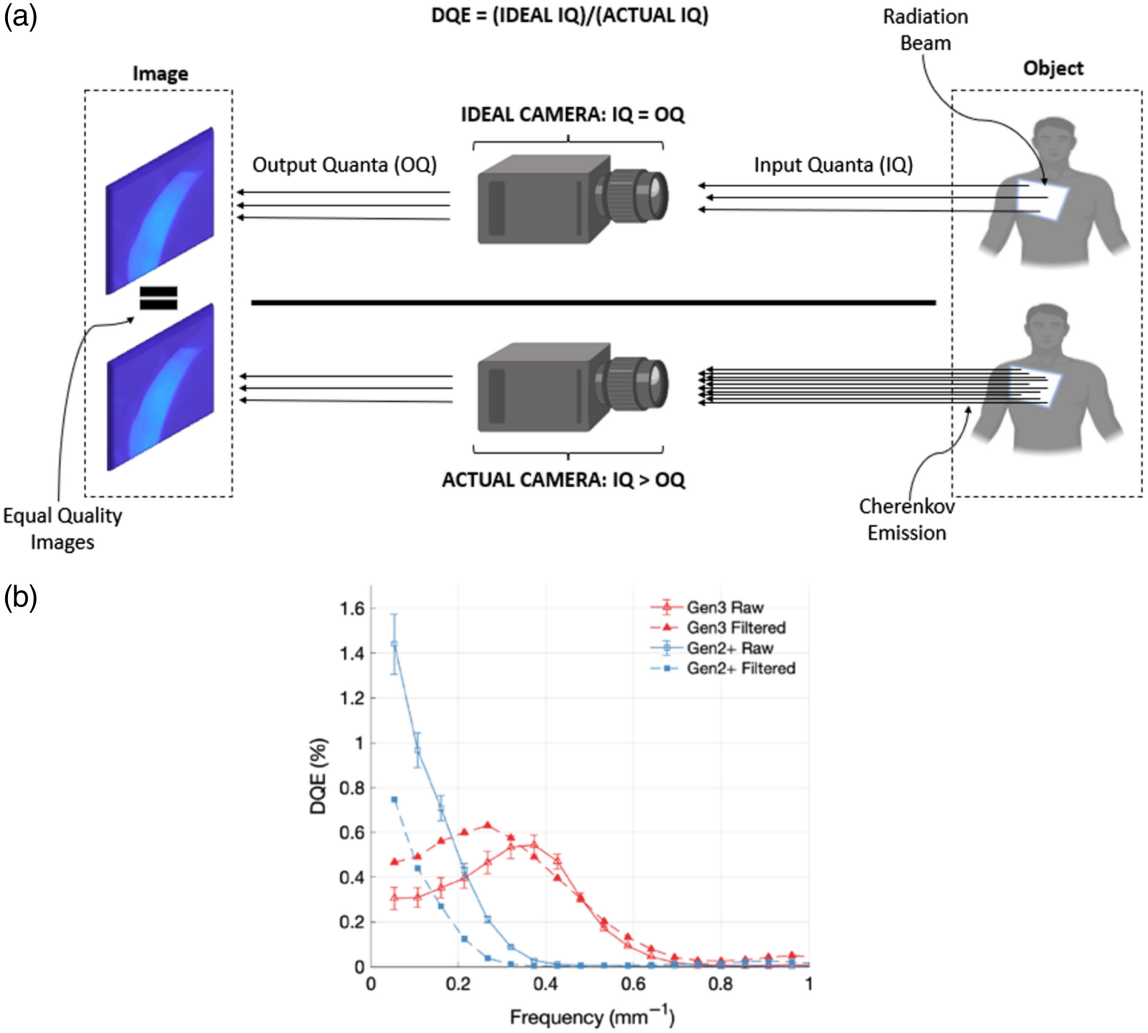
(a) The DQE can be described as the number of quanta used in a perfect imaging system to obtain a certain quality divided by the number of incident quanta required in a nonperfect system to acquire the same image quality. (b) Example of the DQE of various imaging systems as a function of the spatial frequency showing the superior performance of the Gen2+ system in the low-frequency domain. Reprinted with permission from Ref. 50.

The DQE describes the fraction of photons incident on the imaging system required to obtain the same image quality as with a perfect camera system where there are no quanta losses^51^ and can be defined as a function of frequency:^52^^,^^53^

$$\mathrm{DQE}(f)=\frac{d^{2}\cdot [\mathrm{MTF}(f){]}^{2}}{q\cdot \mathrm{NPS}(f)},$$ (6)

where d is the average pixel count, MTF(f) is the modulation transfer function, NPS(f) is the noise power spectrum, and q is the photon fluence. The Gen2+ system was pursued based on the DQE demonstrating the need for this analysis for further image quality developments (Fig. 9).

### 5.3 Postprocessing Techniques

Postprocessing techniques are frequently employed in CT and MRI applications to improve their diagnostic capabilities by suppressing noise artifacts. A popular approach in CT is the alpha (adaptive) trimmed mean (ATM) filter proposed by Hsieh.^54^ Additionally, various postprocessing algorithms exist for digital camera images, such as nonlocal means (NLM), block matching 3D (BM3D), bilateral filter for denoising with preserved edges, and total variation with an L1 mean (TV-L1).^55^^–^^58^ These algorithms were tested on Cherenkov images to determine feasible postprocessing algorithm for improved Cherenkov imaging.^59^

A series of long exposure time cumulative images were used to estimate ground truth data and were compared against the same images exhibiting extensive Cherenkov noise. Based on the peak signal-to-noise ratio, the NLM and bilateral filters exhibit the best performance with the TV-L1 filter also producing comparable results. The BM3D filter performed well at low noise levels but saw a large decline in performance at a lower dose delivered (higher image noise), whereas the ATM filter performed the worst.

Although these approaches have not been implemented in the clinical setting at this point, this study demonstrates the utility of postprocessing in improving image quality. Despite the improvement in cumulative imaging, all filters produced poor results when looking at single-frame images, requiring further research.^59^ Additionally, future studies are likely to combine this analysis with deep learning models to further improve Cherenkov denoising.

### 5.4 Color Map

An often-neglected aspect of scientific communication is the process of displaying data in a way that is equitable and intuitive. Specifically, color maps used for the display of medical images are imperative for the ease of diagnosis and medical incident detection. Cherenkov imaging is no exception.

The incidence of green–red color deficiency in men of European descent has been estimated to be 8% while most female populations see an incidence around 0.5%.^60^ Therefore, it is crucial to display Cherenkov images such that both physicians and patients experiencing some form of colorblindness can interpret the data. Color maps such as turbo and jet should be avoided as these maps contain red and green elements, which are difficult for many color-blind individuals to distinguish. More scientifically based color maps, such as viridis and thermal, maintain a constant gradient in color perception across their range and exhibit a change from light to dark when transformed to a gray scale leading to easier interpretability for someone experiencing total colorblindness.^61^ Clinical implementation of Cherenkov imaging uses scientific color maps to ensure the accuracy of surface dose deposition interpretation.

## 6 Applications

### 6.1 Cherenkov Luminescence Imaging

Because the energy threshold for a beta particle to produce Cherenkov light in water and tissue is ∼0.26  MeV and ∼0.21 to 0.24 MeV, respectively, which is far below the peak emission energy of electrons and positrons for most commercial radiotracers, Cherenkov light can be used to image the delivery of radiotracer agents to particular structures.^62^

**Fig. 10:**
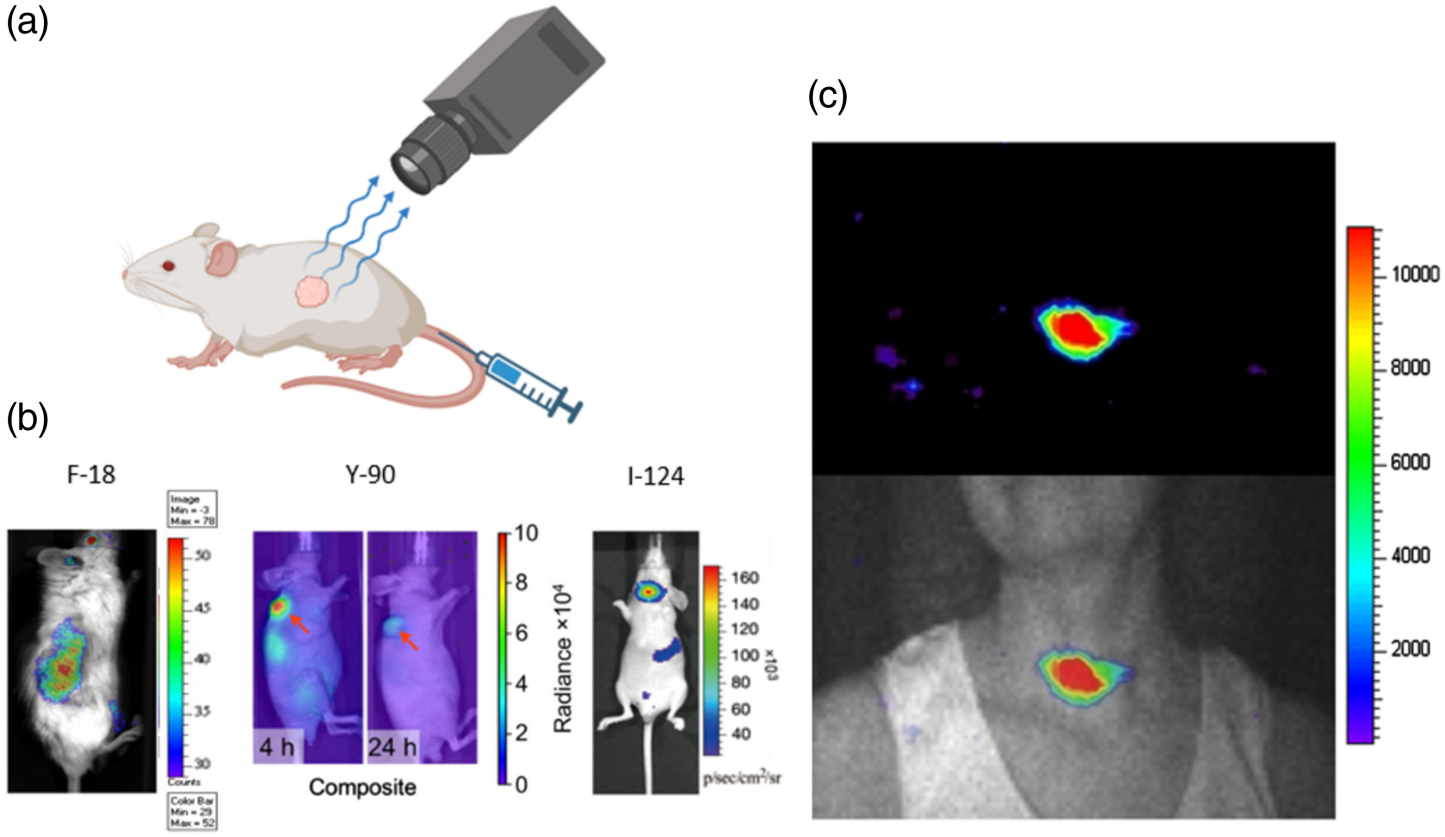
(a) A radioactive drug can be targeted to a tumor. The emission from the source is typically above the energy threshold for charged particles to emit Cherenkov light. If the primary radiation is uncharged, Cherenkov emission is produced when the energy is transferred to secondary electrons. (b) Several different isotopes have been imaged *in vivo* including gamma emitters (F-18 in the form of FDG^64^), beta emitters (Y-90^65^), and positron emitters (I-124^66^). Reprinted with permission from Refs. 64–66. (c) The first Cherenkov luminescence image taken of a human cancer was of a thyroid gland targeted with I-131. Reprinted with permission from Ref. 67.

Cho et al. reported on a method of measuring cherenkov emission from F18-labeled substances using a CCD camera, intending to develop a method for quantitatively imaging beta particles produced in a microfluidic chip.^63^ These chips are used for the synthesis of F18-labeled molecules and the proposed Cherenkov imaging allowed for the spatial detection of points of failure. Additionally, Robertson et al.^64^ reported on the first *in vivo* detection of Cherenkov light from 2-[F18]fluoro-2-deoxy-D-glucose (FDG) with a CCD camera, with resulting Cherenkov images demonstrating tumor uptake of FDG inside the mouse model. Robertson et al. coined the term Cherenkov luminescence imaging (CLI) to describe this planar imaging technique. CLI is a powerful and inexpensive method for testing the uptake of new radiopharmaceuticals (Fig. 10).

Many other radionuclides, including Y90, I131 (beta emitters), and I124, (positron emitter), have been studied and used for CLI procedures and *in vivo* studies.^65^^,^^66^ Furthermore, it has been demonstrated through simulation that alpha chains used for target alpha therapy, such as Ra223, Pb212, and Tb149 produce significant amounts of Cherenkov light for CLI.^68^ Spinelli et al. performed the first human Cherenkov luminescence image, in which the patient received orally a 550 MBq sample I131 24 h before imaging. To detect the weak optical signal, a cooled electron multiplied CCD with QE optimized in the NIR spectrum was used to image the patient in the dark.^69^ Resulting data demonstrated the uptake of iodine into the thyroid with the potential application of measuring the dose delivered to this superficial organ.^67^ Pratt et al. published a clinical study that demonstrated similar results in patients. 96 patients injected with different cancer targeted radiopharmaceutical drugs were imaged with a fibroscope camera system in a dark patient enclosure, producing external body images of ${\mathrm{Na}}^{131}I$, FDG, Ga68-DOTATATE, Lu177-DOTATATE, and RaCl2232 uptake.^70^ These studies demonstrate the feasibility of performing supplemental Chernekov imaging for radiotracer therapies.

Additionally, 3D Cherenkov images of *in vivo* radioactive sources are attainable, coined Cherenkov luminescence tomography (CLT). Using several 2D CLI images at different angles, the CLT image can be reconstructed inversely using the diffusion equation employed in bioluminescence tomography.^62^^,^^71^ Another 3D reconstruction method proposed by Spinelli et al. removes the need for multiple view angles using spectral information obtained through taking several images at one view angle with different wavelength bandpass filters, which is sufficient for 3D reconstruction of the source.^72^

Although difficult to apply to *in vivo* patient studies due to increased attenuation through human skin, clinical studies have demonstrated the use of CLI in surgical guidance and cancer screening. A clinical trial involving the use of CLI for tumor margin determination in breast conserving tumor resection surgery demonstrated promising results using 5  MBq/kg F18-FDG. Resected tissue surrounding and including the tumor was imaged in a light tight environment with the optical margins showing excellent agreement with the histologic margin distance.^73^^,^^74^ A similar clinical trial measured excised prostate tissue from patients injected with 100 MBq Ga68-PSMA, showing the localization of Ga68 uptake. In both studies, exposure to the medical staff was low. For cancer screening, CLI can act as an alternative to PET, the current clinical gold standard. A 2014 study reported on the injection of F18-FDG into patients who were first imaged with PET before having CLI performed in a dark room with a CCD camera. The nodal uptake signal recorded using CLI showed excellent agreement with the PET images.^75^^,^^76^ Although clinical applications of CLI using radiopharmaceutical agents are still in the development stage, this field laid the groundwork for the advancements in external beam Cherenkov imaging and Cherenkov excited luminescence imaging.

### 6.2 Imaging Dose with Cherenkov and Scintillation

**Fig. 11:**
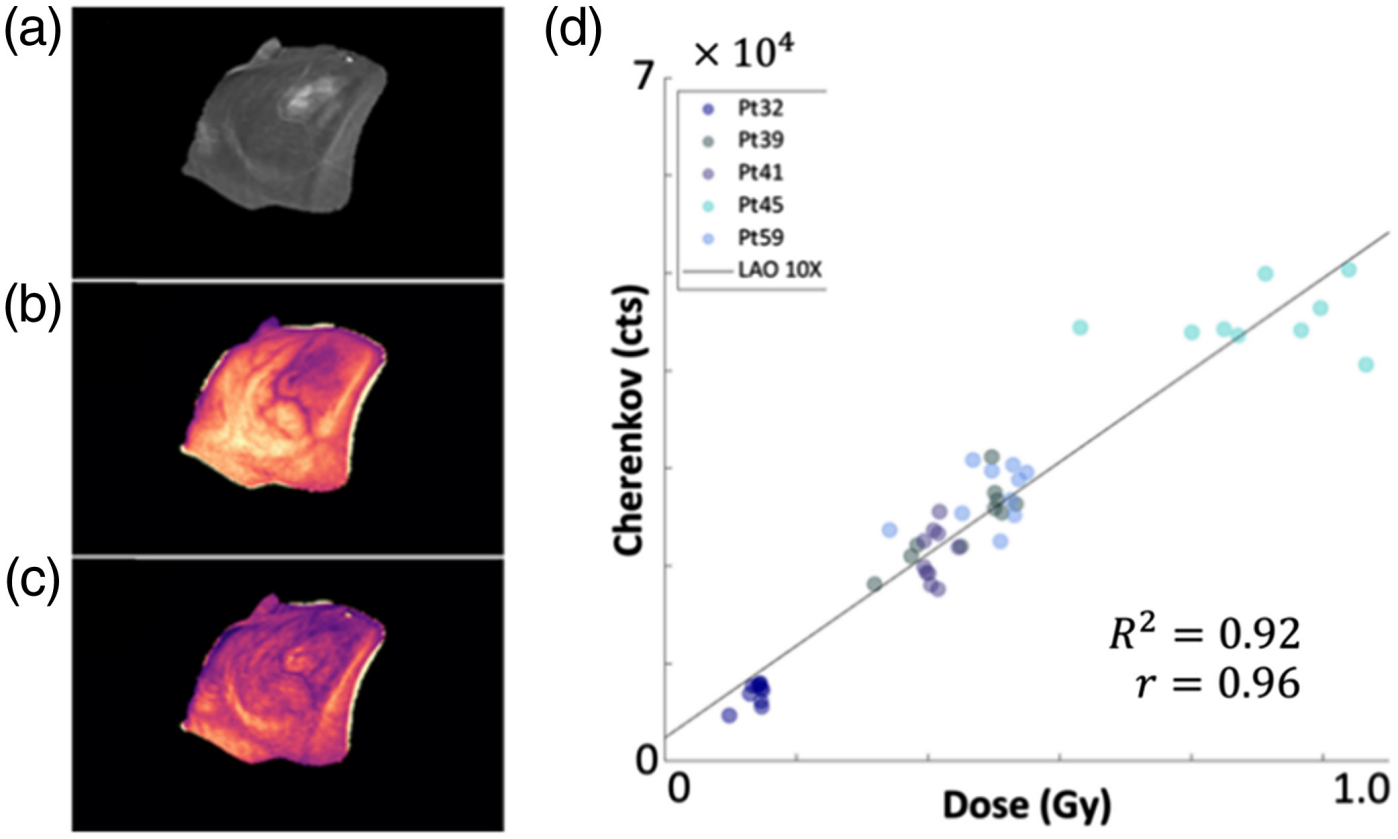
(a) CT, (b) uncorrected Cherenkov, and (c) CT corrected Cherenkov image data for breast radiotherapy treatment. (d) During these treatments, the CT corrected image data were found to have good linearity with dose. Reprinted with permission from Ref. 79.

Cherenkov light emission imaging allows for both real-time imaging of the radiation field^5^^,^^22^ and real-time dosimetry for patient treatments using photon^6^ or electron beams.^77^ During patient treatment, the use of Cherenkov light emission imaging allows clinicians to visualize the radiation field and MLC motion and ensure plan delivery. This system has shown that high-intensity regions within a composite Cherenkov image correspond to skin reactions on the patient^5^ and is capable of detecting stray radiation deposited in patients’ eyes during radiotherapy treatments to the brain.^78^ Further clinical implementation of Cherenkov light emission imaging has also led to the detection of treatment delivery issues that were not prevented using standard QA and mitigation strategies.^8^ Cherenkov light emission imaging has also been investigated for use as a quality assurance (QA) technique in high dose rate brachytherapy where sources are imaged in water phantoms, and the Cherenkov emission is quantified to simultaneously measure dose distribution, source strength, and source position (Fig. 11).^22^^,^^80^

**Fig. 12:**
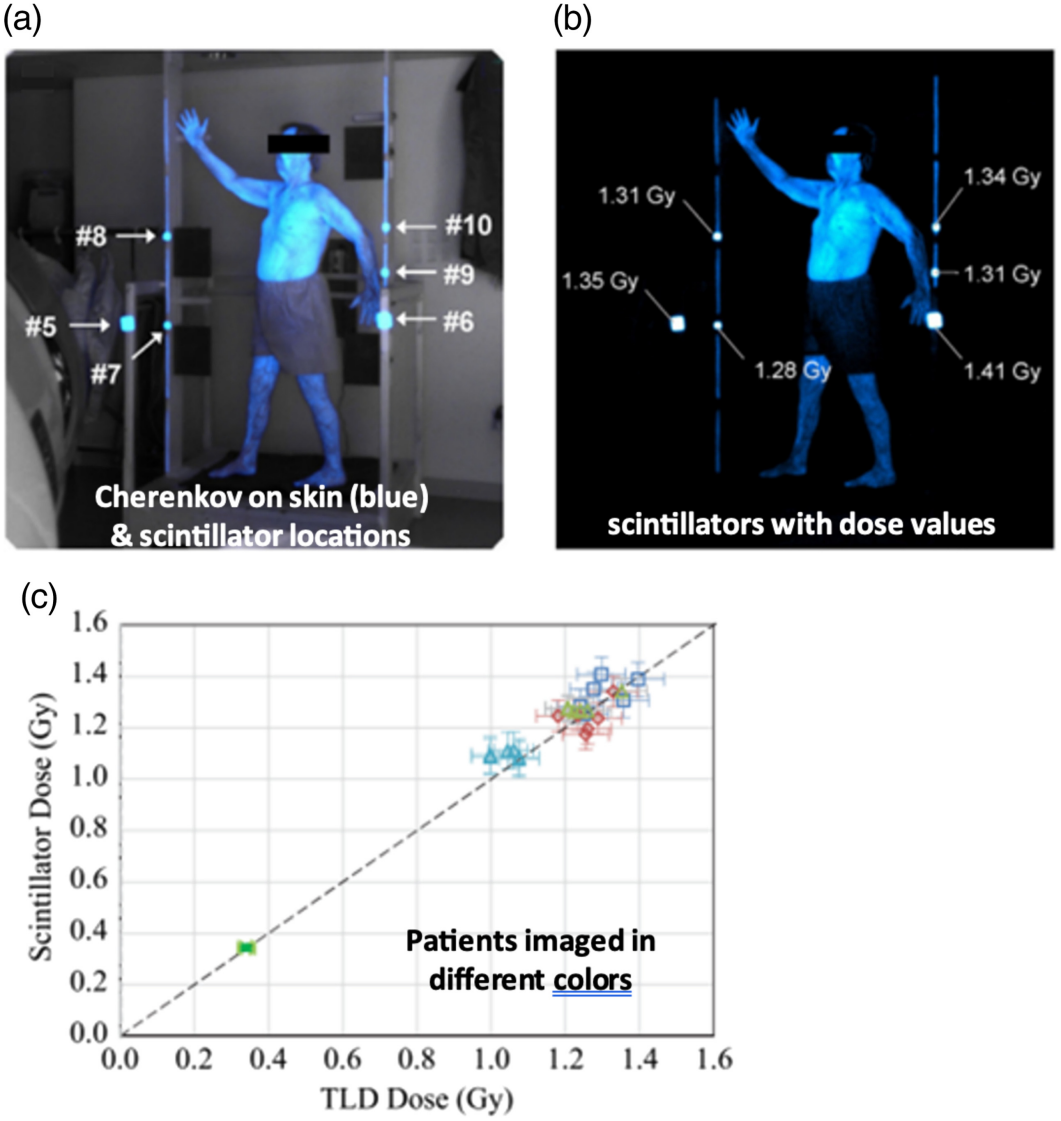
Images of a patient undergoing TSET treatment with scintillators (a) with and (b) without the background signal. (c) Scintillator signal and dose were found to be linear during TSET treatments across different patients imaged. Reprinted with permission from Ref. 77.

**Fig. 13:**
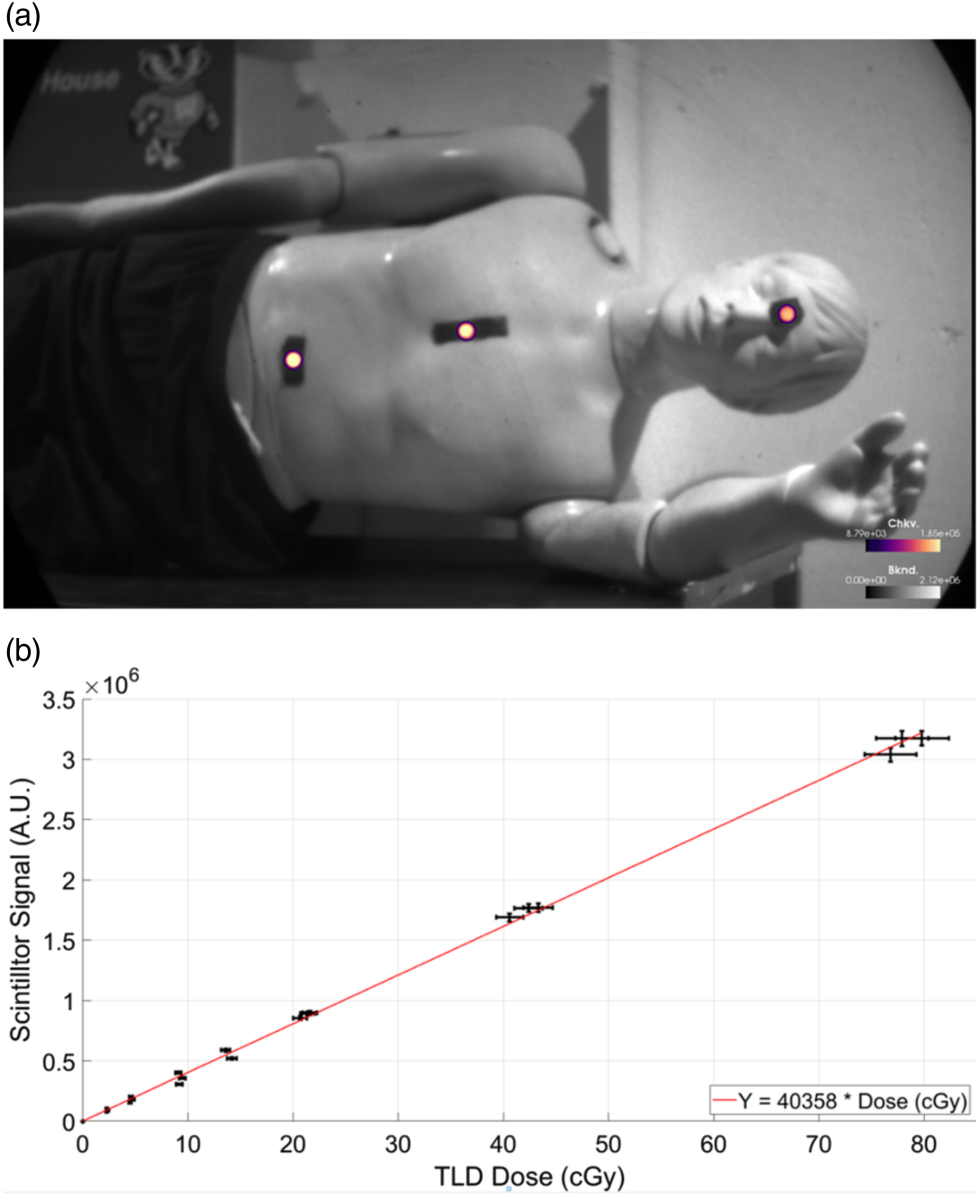
(a) An anthropomorphic phantom with scintillators on the abdomen, chest, and forehead to monitor dose homogeneity during TBI treatments. (b) Under TBI conditions, the scintillators continued to have a linear relationship with dose. Reprinted with permission from Ref. 84.

To perform real-time dosimetry, a time-gated camera has been used in conjunction with CT scans^79^ or fast-response plastic scintillators.^6^^,^^81^ The Cherenkov signal produced in patients is attenuated and affected by local tissue optical properties.^82^ By imaging scintillators or correcting Cherenkov light images with CT scans, confounding factors, such as underlying patient anatomy or patient immobilization structures are eliminated as the Cherenkov light is no longer the sole signal used for dosimetry. Two areas of note where imaging of scintillators has been tested are total skin electron therapy (TSET)^83^ and total body irradiation (TBI).^84^ In both TSET and TBI, the patient is placed at a much further distance from the radiation source^85^^,^^86^ and, by extension, the camera (Figs. 12 and 13).

When implemented for TSET, the dose response to scintillators was found to be linear with the other dosimeters used (TLDs and OSLDs), and signal variation between scintillators was reported as 0.3%±0.2%.^83^ The standard error of the cumulative dose from the scintillators, compared to other scintillators, was 5%.^77^ In TBI experiments, the imaging of scintillator dose has continued to show good agreement with other dosimeters—achieving repeated agreement with TDLs to within 2.7% and good linearity ($R^{2}=0.996$).^84^ For contrast, other experiments using inorganic scintillators were able to achieve repeatability and maximum deviation of 0.26% and 0.5%, respectively. It is worth mentioning that these experiments used a different detection scheme with optical fibers carrying the scintillator signal to a photon counter.^87^

In both cases, imaging scintillator dose provides instant dosimetric feedback to clinicians, during the treatment as a means of viewing dose as it is delivered and as a cumulative dose for the treatment fraction. There is also a necessity to correct scintillator signals with the inverse-square law as scintillators placed on the patient may vary in distance from the camera used to image them. An example is the case where a scintillator is placed on the patient’s abdomen and visible in the same image frame is a scintillator on the patient’s forehead. In this case, the camera may be physically closer to the scintillator on the patient’s abdomen, meaning that to properly compare scintillator signals between the abdomen and forehead, corrections must be made. Room lighting is also a known issue but is more prevalent in TBI cases. To correct for the presence of room lights the images are background subtracted. This improves the image quality but as TBI has lower dose rates, the scintillators produce less signal in each captured frame that requires special consideration for the TBI implementation, such as brighter, wider, or thicker scintillators, in order to produce more signal per frame.

In both the TSET and TBI implementations of Cherenkov light imaging, there are special considerations for the angles between the scintillator and the camera (scintillator-camera angle) and between the linear accelerator treatment head and the scintillator (source-scintillator angle). It was found that to maintain a radiant energy fluence precision within 1% that both the scintillator-camera angle and the source-scintillator angle need to be held below 50 deg for TSET.^77^ In TBI cases, to maintain precision within 5% the source-scintillator angle is to be held below 30 deg.^84^ In either case, a reason to reduce the source-scintillator angle is a result of dose-buildup effects where as the source-scintillator angle is increased the radiation beam passes through more of the scintillating material that will produce varying amounts of signal.^77^

An additional effect of imaging and measuring Cherenkov in reference to scintillator dosimetry is that it can become a background signal, which then confounds the scintillator signal. When using time-gated cameras, the Cherenkov signal is often significantly less than the scintillator signal that results in a background term several orders of magnitude lower than the scintillator signal. In TBI cases, where a transparent acrylic spoiler is present consideration must be made for the Cherenkov light produced within the acrylic, which is then detected by the camera.^84^ Other applications of scintillator dosimetry that utilize optical fibers to transmit the scintillator light to a photon counter also encounter a similar background signal where Cherenkov is produced within the optical fiber. In these cases, the Cherenkov light should also be removed, which can be done through a variety of means including background subtraction, simple filtration, and chromatic removal.^88^^,^^89^

### 6.3 Cherenkov and Surface-Guided Radiotherapy

Cherenkov imaging commercially can be considered a subset of the larger efforts in what is called surface guided radiation therapy (SGRT).^90^^–^^92^ There are different terms used to describe this, driven by different vendors, but the essence is that optical imaging of the patient’s surface is utilized to guide radiotherapy deliver and decisions.^93^ The standard systems project light onto the patient, and image the projections, recovering some surface topology and with substantial software processing calculate the locations of all relevant parts of the patient in real time. This surface mapping provides the therapy team with a recording of the location and the ability to utilize this surface to ensure that the patient position is matched with the CT simulation scan so that delivery will be accurate. Moreover, day to day positioning in fractionated radiotherapy is improved by this tool, given how much time a patient requires to find the same position in each fraction. Cherenkov imaging has been integrated into SGRT by one vendor, and the combined surface mapping and Cherenkov display of beam delivery on the surface provide an enhanced SGRT feedback to the therapy team. This innovation is still embryonic but the utilization of this for human delivery verification will likely be important tool in the suite of the therapists, as well as for investigation of variations that require further information.

### 6.4 FLASH Radiotherapy with Cherenkov Imaging

FLASH radiotherapy employs an ultrahigh dose rate (UHDR) of >40  Gy/s rather than conventional radiotherapy delivery of ∼0.1  Gy/s and has been seen to preferentially harm cancerous tissue over normal tissue in preclinical and clinical studies. Due to its ∼1000× higher density of energy per unit time when compared to conventional radiotherapy, increased Cherenkov light will be produced in UHDR conditions, allowing Cherenkov cameras to be a potential alternative to previously used, energy-dependent dosimeters.^94^ Additionally, just as with conventional therapy, there is potential for imaging Cherenkov light emitted from a patient for image guidance, as long as the camera’s temporal speed is on the order of FLASH treatments.^94^

## 7 Summary

Several key innovations have come together to make Cherenkov imaging possible, which are summarized below. These together enable the science of how to image a single-photon level within a lighted room, and the medical science of what can be seen with the Cherenkov images. The potential promise and remaining challenges are critical to review, as listed below.

### 7.1 Summary of Technology Advances

There have been several key technological advances that have been central to the realization of Cherenkov imaging in clinical radiotherapy. The first of these has been the concept and implementation of triggering the camera acquisition by remote sensing of stray radiation. This single invention allowed the cameras to be utilized for time-gated imaging without contact to the linear accelerator. The second major innovation was in the realization that time-gated imaging was necessary and that with the low duty cycle of most linacs, this allowed for substantial (1000×) removal of background light from the image. The third major innovation was in image processing, utilizing both temporal and spatial filtering (primarily median filtering) to remove spurious events in the camera that have low prevalence at any given pixel and at any given time. The fourth major event has been in wavelength filtering, recognizing that the Cherenkov coming from patients is in the 650 to 950 nm range, and therefore, allowing removal of any extra signal outside this range. The fifth innovation was in the simple process of acquiring dynamic background images, with background image acquisition when the linac is not delivering radiation, and online subtraction of this from the time-gated images. This latter process is achieved at video rate through processing (Table 1).

**Table 1:** Key technological innovations that have advanced the capabilities of Cherenkov imaging in radiotherapy.

Technological innovation	Improvement
Triggering off of stray radiation	Essential to avoid electrical contact to linac
Time-gated intensifier	≈1000× background removal of because of 1/1000 duty cycle
Spatial and temporal filtering	Essential to remove overwhelming noise
Optical filtering	≈2× reduction in background light signal
Background signal subtraction	≈2 to 4× reduction in background

### 7.2 Summary of Medical Advances

The medical utility of Cherenkov is clearly the predominant motivation for the development of Cherenkov imaging, to be used as a device for visualization of the radiotherapy beam. The areas of utility are broken down in Table 2. They range from real-time visualization, which has value for the radiotherapy team to see the treatment as it happens and to be able to use this as a real-time tool for visualization. The next value is in the time-integrated Cherenkov, which can then show the total treatment and be used for 2D image comparisons, such as day to day consistency checking or for comparison to surface dose estimates from the treatment plan. The recorded delivery sequence has inherent value because the permanent record can then be used for review in the case of an investigation or an incident. The combination of Cherenkov imaging with other components will also lead to potential added value, one of these being the integration with SGRT and the other being imaging of scintillator reflectors on the tissue. These each have unique and separate value, and themselves are in varying stages of commercial deployment. Finally, the original studies of Cherenkov focused on water phantom imaging for linac quality assurance (QA) testing and as the cameras get better these may be more widely utilized. Beyond this though, there could be utility in verification of complex treatment plans through imaging of the plan delivery in a water tank or on an anthropomorphic body phantom.

**Table 2:** Signals that can be observed with these imaging systems have a range of values as listed.

Signal observed	Value
Real-time Cherenkov on patient	Delivery verification
	Delivery stop during incidents
Cumulative image on patient	Day-to-day consistency
	Surface dose comparison
Recorded delivery sequence	Review of delivery
	Chart check
	Investigation of incidents
Combined Cherenkov and patient position	SGRT with Cherenkov
Scintillator reflector imaging	Total surface dose at set points on patient skin
Cherenkov from phantoms and water tanks	Linac and plan QA

### 7.3 Summary of Science Advances

Perhaps the single most important science advance in Cherenkov imaging has been the realization that high-resolution single-photon imaging can be achieved within ambient room lighting. The technological solutions listed above in Sec. 7.1 have made this possible, but it now opens up the idea that light can be sampled from patients during radiotherapy. Additional innovations have been explored in the idea that optical sensors maybe added to the patient to sense things like tissue destruction (ref), oxygen or pH within the tissue. Engineered optical contrast agents that have long-lifetime emission properties may become important parts of sensing in radiation therapy, either for particular metabolism features, or more simply for *in vivo* radiation dose delivered inside the tissue (Table 3).

**Table 3:** There are a range of current and future possible innovations listed with their utility and the origin of the signal that can be captured.

Medical/biomedical innovations in utility	Signal
Single-photon imaging capturing Cherenkov	Cherenkov intensity
Tissue destruction captured by diffusion of tattoo ink	Ink luminescence intensity^95^^,^^96^
Oxygen sensing from within irradiated tissue	Phosphorescence lifetime^97^
Metabolite sensing from within irradiated tissue	Luminescence from engineered sensors
Retina dose sensing	Cherenkov out of pupil
Short-wave infrared imaging	Short-wavelength Cherenkov emission^98^

### 7.4 Remaining Challenges

The value of Cherenkov imaging is still actually emerging as adoption into clinical practice is just emerging at this point. However, there are several unexplored or undeveloped areas of Cherenkov that could have value. These can be categorized several ways, but here are outlined in terms of (1) the physical properties of Cherenkov un-utilized at this point, (2) improvements to sensors that could improve the detection and use, (3) improvements in the biological interpretation of the images, (4) improvements in the dosimetric interpretation of the images, and (5) advances in automation that might incorporate Cherenkov. Each of these is listed in Table 4, with a range of the unique features that have not been fully explored or exploited yet. These are speculative but the listed features are unique and as such could have value that is unknown at this point.

**Table 4:** Future potential of Cherenkov could be augmented if currently unexplored features are exploited.

Potential remaining	Feature
Utilization of unique Cherenkov physical properties	Polarization
	Spectrum
	Time sequence
	Unique origin in soft-electron collisions
	Angular sensitivity
	Quantum nature of emission
Improved technological approach (sensors and cameras)	Higher sensitivity
	Lower sensor noise
	Lower background sensitivity
	Lower noise/artifacts
	Angular sensitivity
	Coincidence detection utilization
	Lower complexity
	Lower cost
Biological-Cherenkov interactions	Use of biological features for fiducials or interpretation of treatment accuracy (vessels or erythema)
	Molecular sensors that are excited by Cherenkov
	Molecular therapeutics that are activated by Cherenkov
Cherenkov to dose interpretation	Explicit correction for attenuation effects
	Implicit surrogates to correct for attenuation
Automation of use	Designing processes that utilize the signals without the need for human intervention

#### 7.4.1 Potential for machine learning

The use of machine learning has revolutionized the field of medical imaging in recent years with applications spanning from denoising to clinical diagnosis.^99^ While there have been attempts to implement machine learning into the Cherenkov imaging workflow, the applications have yet to be fully realized in this space making it fertile ground for development. As mentioned previously, Cherenkov images suffer from excessive amounts of noise. A machine learning approach may be capable of overcoming this noise, producing images more indicative of the spatial dose spread. Such an approach may employ a U-Net or GAN architecture to denoise single-frame images using cumulative images as the ground truth.

In addition to denoising applications for deep learning in the field of Cherenkov imaging, neural networks can also be trained to detect medical incidents based on the EBRT Cherenkov image data. The difference between the spatial distribution of surface dose measured using Cherenkov imaging, and the planned surface dose can be indicative of improper radiation dose delivery. Since a physician may not have time to review every treatment, a machine learning algorithm trained on medical incident data could greatly improve the patient’s treatment plans for future treatment fractions. This is necessary because traditional metrics (i.e., Dice similarity) have blind spots that machine learning can detect.

## Data Availability

Data sharing is not applicable to this article, as no new data were created or analyzed.
